# A Review of Recent Advances in 3D Bioprinting With an Eye on Future Regenerative Therapies in Veterinary Medicine

**DOI:** 10.3389/fvets.2020.584193

**Published:** 2021-02-16

**Authors:** Colin Jamieson, Patrick Keenan, D'Arcy Kirkwood, Saba Oji, Caroline Webster, Keith A. Russell, Thomas G. Koch

**Affiliations:** Reproductive Health and Biotechnology Lab, Department of Biomedical Science, University of Guelph, Guelph, ON, Canada

**Keywords:** bioprint, tissue engineering, regenerative medicine, 3D print, additive manufacturing, veterinary

## Abstract

3D bioprinting is a rapidly evolving industry that has been utilized for a variety of biomedical applications. It differs from traditional 3D printing in that it utilizes bioinks comprised of cells and other biomaterials to allow for the generation of complex functional tissues. Bioprinting involves computational modeling, bioink preparation, bioink deposition, and subsequent maturation of printed products; it is an intricate process where bioink composition, bioprinting approach, and bioprinter type must be considered during construct development. This technology has already found success in human studies, where a variety of functional tissues have been generated for both *in vitro* and *in vivo* applications. Although the main driving force behind innovation in 3D bioprinting has been utility in human medicine, recent efforts investigating its veterinary application have begun to emerge. To date, 3D bioprinting has been utilized to create bone, cardiovascular, cartilage, corneal and neural constructs in animal species. Furthermore, the use of animal-derived cells and various animal models in human research have provided additional information regarding its capacity for veterinary translation. While these studies have produced some promising results, technological limitations as well as ethical and regulatory challenges have impeded clinical acceptance. This article reviews the current understanding of 3D bioprinting technology and its recent advancements with a focus on recent successes and future translation in veterinary medicine.

## Introduction

**Figure 1:**
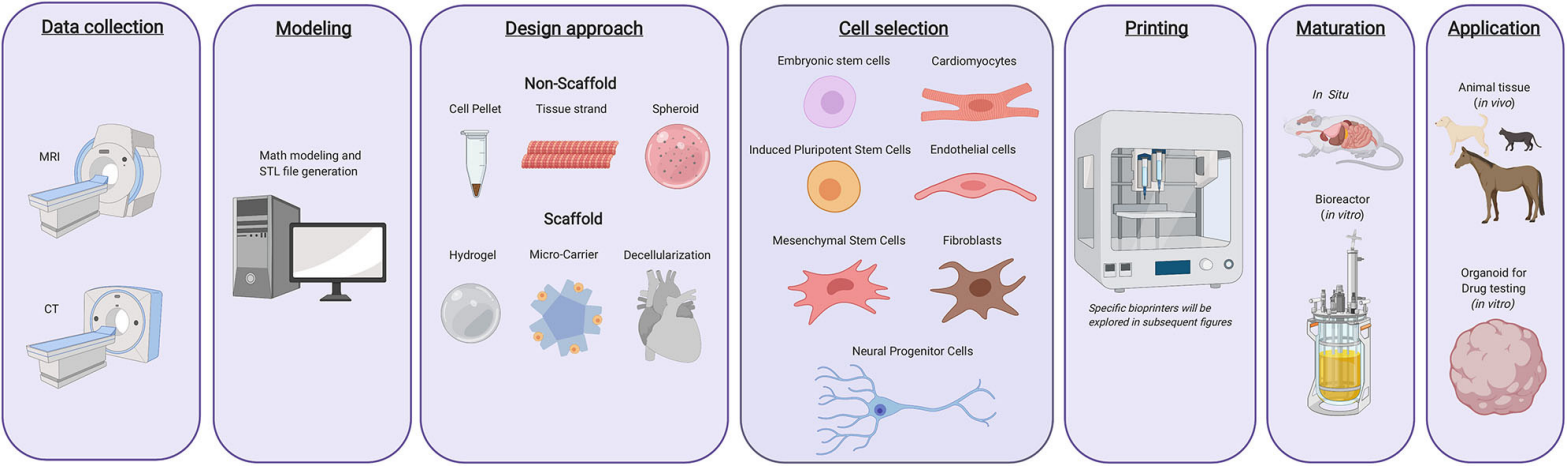
Typical 3d bioprinting workflow. Tissues or organs are first imaged using a variety of techniques as to generate a guide that can be used while printing the desired constructs. The design approach is then selected, which includes biomimicry, self-assembly, or mini tissues. Materials and cells sources are then selected based on the desired tissue type, form, and function. Once these components have been selected, they are integrated into a bioprinting system and the 3D construct is generated. Following printing, the constructs can either be used immediately for *in vitro* purposes, *in vivo* transplantation, or can be matured further in a bioreactor before being utilized for other applications.

3D bioprinting is a rapidly evolving industry that has the potential to reshape regenerative medicine (1). 3D bioprinters use bioinks comprised of living cells and biomaterials to generate 3D printed tissues. This process follows a workflow comprised of computational modeling, bioink preparation, bioink deposition, and subsequent maturation of printed products (Figure 1) (2). Bioprinting is a versatile tool able to produce a wide range of tissues and organs. Access to bioprinted organs could help resolve the current human organ shortage crisis. In combination with advances in tissue engineering, these technologies could also aid in the treatment of several conditions within veterinary medicine including equine bone fractures, articular cartilage repair, or the generation of more accurate disease models (3). Its prospective applications have fueled the expansion of research and commercial efforts lending to significant advancements in the field. As a result, the 3D bioprinting industry is predicted to be valued at $1.82 billion USD by 2022 (4). However, despite recent innovations, 3D bioprinting must overcome significant technological, ethical, and regulatory challenges before it can be implemented in clinical practice (5).

The driving force behind recent advances in 3D bioprinting has been its utility in human medicine. However, bioprinting has significant implications for veterinary medicine as well. Research efforts targeting human applications have utilized companion animal models to investigate the safety and efficacy of bioprinted tissues. Their findings have provided background for human clinical trials and helped characterize the therapeutic utility of 3D bioprinting in veterinary science (6). This has provided the groundwork for veterinary research efforts and will likely expedite future veterinary translation. Furthermore, preliminary *in vivo* analyses have produced promising results in support of future veterinary translation (7–11). This review will focus on 3D bioprinting in veterinary medicine and outline the current understanding of 3D bioprinting technologies, its applications, and the challenges it is likely to face as this industry strives for clinical acceptance.

## Cellular Component of Bioink

Bioinks are inks comprised of cells and other biomaterials and are defined by their printability and cytocompatibility. Their printability influences shape fidelity and mechanical stability, whereas cytocompatibility dictates cell viability, migration, proliferation, differentiation and subsequent tissue formation. Bioink properties are chosen to compliment bioprinter type as well as bioprinting approach according to the desired tissue. Further, printer type and bioprinting approach must also be considered when choosing the appropriate bioink. Although somatic cells such as chondrocytes, fibroblasts, and cardiac myocytes have been used in 3D bioprinting, most applications rely on the inclusion of stem cells to facilitate *de novo* tissue development (12–14). Bioprinting exploits the ability of these cells to undergo self-renewal and directed differentiation to control tissue development and ultimately generate bioprinted tissues. Stem cells used in bioprinting can be separated into two categories: pluripotent and multipotent stem cells.

Pluripotent stem cells can, in principle, differentiate into any cell type in the body. There are two types of pluripotent stem cells: embryonic stem cells (ESCs) and induced pluripotent stem cells (iPSCs). ESCs are derived from the inner cell mass of the blastocyst. These cells require minimum genetic manipulation and possess a low risk of subsequent tumor formation. However, there exist ethical concerns regarding their use since their isolation is associated with the destruction of an embryo, albeit commonly a discarded embryo from an *in-vitro* fertilization clinic (15, 16). The use of iPSCs avoids ethical conflicts for they can be generated from adult cells. However, reprogramming cells to a pluripotent state can be difficult to achieve and maintain (17, 18).

Multipotent stem cells are adult stem cells that, under normal physiological circumstances, only develop into a discrete number of cell types (16). These cells can be obtained from a variety of tissues with little ethical conflict. Traditionally, it was believed that adult stem cells were only capable of giving rise to progeny related to their tissue of origin; however, studies have found that these cells can be transdifferentiated into additional cell lineages. Two main advantages of adult stems are that transdifferentiation is more efficient than reprogramming iPSCs, and their use is associated with a decreased cancer risk compared to iPSCs (19). However, procurement of these cells can require invasive procedures such as bone marrow aspiration or liposuction-based techniques. Nevertheless, iPSCs and mesenchymal stromal cells (MSCs) are currently the most used stem cell populations in 3D bioprinting (1).

Recent advances in 3D bioprinting has led to the introduction of alternative bioink formulations including those that use exosomes in replace of stem cells. Exosomes are secreted membrane-bound extracellular vesicles that contain protein, DNA and/or RNA from their parental cells. These vesicles can modulate cell growth and development, and thus, have shown promise in 3D bioprinting either alone or in combination with stem cells. Bioprinted exosomes can create targeted microenvironments that can help correct aberrant cellular activity and direct development of adjacent host tissue (20). Exosomes isolated from bone marrow MSCs printed within a 3D scaffold at the sites of osteochondral defects in white rabbits were shown to reduce cartilage mitochondrial dysfunction, attenuate chondrocyte degeneration, and stimulate osteochondral defect repair (21). Incorporation of additional biomaterials such as decellularized ECM (dECM) and extracellular scaffolds have also been used been in order to direct tissue development, improve cell viability and ultimately the success of bioprinting. These topics are covered in the following section.

## 3D Bioprinting Approach and Bioink Selection

While stem cells are generally utilized to provide the cellular components of 3D bioprinted tissues, adjacent architecture and support are still required for successful tissue generation. Generation of tissue architecture is accomplished via one of two methods: scaffold-based bioprinting, which utilizes an exogenous scaffold to provide mechanical support during tissue development, or scaffold-free bioprinting, which exploits the intrinsic ability of cells to generate adjacent tissue architecture (22). As a result of the characteristic differences between scaffold-based and scaffold-free bioprinting approaches, each require specific bioinks to accomplish successful tissue generation.

### Scaffold-Based Bioprinting

Scaffold-based approaches use biomaterials to create a temporary structure that supports cell attachment, proliferation, and subsequent tissue formation. This technique utilizes a biomimicry approach where individual components of a 3D construct are generated to mimic those properties of a native tissue or organ (23). Scaffold-based bioprinting is more economical and scalable due to its lower cell density requirements, and it provides higher resolution when compared to scaffold-free techniques. However, the presence of exogenous scaffolds can reduce cell-to-cell interactions and degrade into toxic byproducts over time (24).

Scaffold-based bioprinting utilizes hydrogels, microcarriers, or dECM-based bioinks. The most common scaffold-based bioprinting approach uses exogenous 3D constructs of hydrogels. Hydrogels are used to encapsulate cells and other biological molecules that are subsequently seeded into scaffolds. They can be manipulated into any shape, size or form, and have the ability absorb up to a thousand times their dry weight. These characteristics allow the hydrogel to act as a cell carrier and provide flexibility during its production (25). Hydrogels can be derived from natural sources such as gelatin, fibrin or collagen, or synthetically derived (examples include polyethylene glycol and Pluronic® F-127) (26). Currently, Alginate is the most often used material in 3D bioprinting processes; however, synthetic sources have gained recent attention due to their lower batch-to-batch variation and higher level of control over factors such as degradation and mechanical stability (27, 28). Hydrogels derived from a mixture of natural and synthetic sources are called hybrid bioinks. Hybrid bioinks enable researchers to utilize the benefits of multiple sources to customize a bioink that is specifically tailored to their intended application (29). Post-bioprinting, hydrogels are cross-linked via thermal, chemical or physical methods to increase their structural integrity. These methods as well as additional hydrogel characteristics have been reviewed elsewhere (28).

Supportive matrices known as microcarriers can be added to bioink formulations to increase cell density and provide structural support. Microcarriers can be comprised of synthetic or natural materials such as plastic and glass, or cellulose and gelatin, respectively (30). These structures possess a small spherical shape containing interconnected pores ranging from 60 to 400 μm in size (30, 31). These features promote efficient cell adhesion, robust cell proliferation and differentiation by modulating cell shape and organization (30). Microcarriers serve as substrates for anchorage-dependent cellular adhesion and preserve the phenotypic stability of printed cells ^21^. Their spherical structure improves the transfer of gases and nutrients ultimately leading to a larger surface area for viable cell attachment (32). However, limitations of microcarriers include limited scalability, degradation of some microcarriers can produce toxic products, they require a complex detachment system, and their adhesive character may result in nozzle clogging (30).

**Figure 2:**
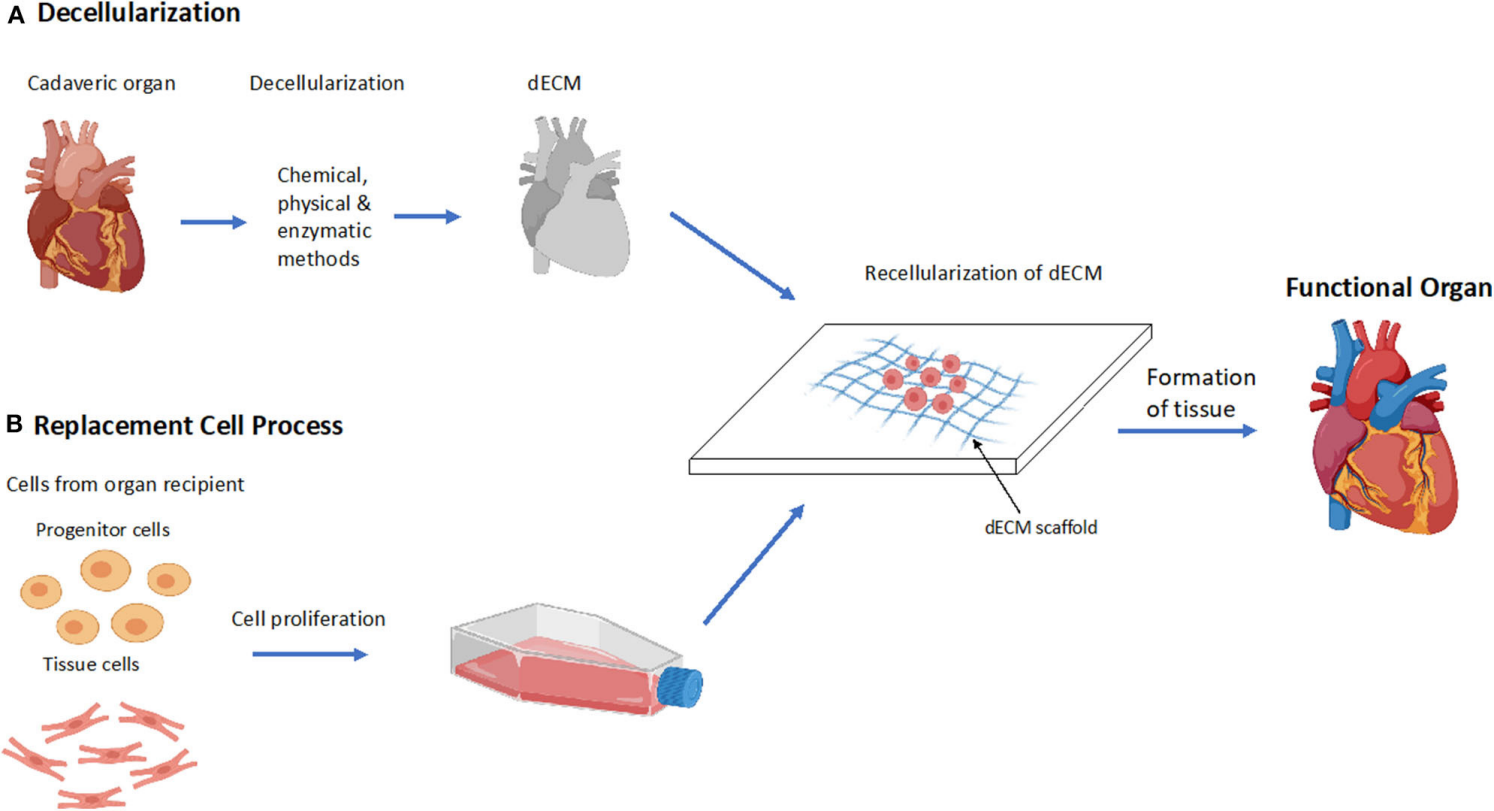
Decellularization process to form a functional organ. **(A)** Overview of the process of decellularization of a cadaveric heart to a functional heart. Decellularization of cadaveric organs followed by the recellularization of the dECM scaffold which forms functional tissue. **(B)** Overview of the stage's replacement cells undergo during the process of recellularization. Cells are extracted from the recipient of the organ and are cultured and proliferated, then inserted into the dECM where it forms a functional tissue.

Decellularization is a novel method used in tissue engineering to create scaffolds comprised of biologically relevant components of the ECM (33). Decellularization involves the removal of the cellular components of a donated organ while retaining its ECM components (34). The dECM provides site-specific mechanical and biochemical interactions that guide cell adhesion, proliferation, and differentiation (Figure 2) (34, 35). Decellularization is performed via chemical, physical or enzymatic methods outlined in Table 1. It has conventionally been seen as an alternative to 3D bioprinting; however, recent efforts have converged these two technologies by utilizing dECM as a bioink. dECM can be modified to create a soft, gel-like material that can be loaded into a 3D bioprinter (39). Due to the retention of native ECM components, dECM bioinks aid in tissue regeneration and cell stabilization, and can help facilitate favorable tissue organization and remodeling (39). Of importance, cell-laden dECM bioinks have been shown to increase the vasculature of printed tissues thereby helping overcome conventional barriers associated with vascular integration (39). Although dECM bioinks can mimic native tissue environments and provide construct stability, dECM bioink alone does not contain the required mechanical strength needed to develop load-bearing tissues and therefore require scaffolds for additional support. Further, due to the requirement of donated organs to facilitate dECM isolation, its use lacks scalability (39).

**Table 1:** Current methods of decellularization and their advantages.

**Method**	**Method subtype**	**Materials used**	**Advantages**	**References**
Chemical			Cost effective, efficient, most commonly used method	(36, 37)
	Acids	CH3COOH		(37–39)
	Bases	NH_4_OH, Ca(OH)_2_, NaOH, Mg(OH)_2_		(36–39)
	Hypotonic/Hypertonic solutions	Tris-HCL		(36–39)
	Ionic detergents	SDS, Trition X-200, SDC		(36, 37, 40)
	Non-ionic detergents	Triton X-100		(38, 39, 41)
	Zwitterionic detergents	CHAPS, SB-10, SB-16		(36, 37, 42)
	Chelating agents	EDTA		(36, 37, 40)
Physical			Cost effective, supplies readily available, low risk of harming ECM, minimizes amount of chemicals used	(36, 37)
	Freeze/thaw	Liquid nitrogen		(37, 40)
	Force/pressure			(42)
	Electroporation	Electric field oscillation		(36, 37, 42)
	Agitation			(42)
	Sonication	Ultrasonic waves		(37, 42)
Enzymatic			Disrupts the interactions between cells without harming ECM, reduces time exposed to chemicals	(37, 42)
	Protease	Trypsin, Dispase II		(36, 37, 39, 43, 44)
	Exo/endo nucleases	RNase, DNase		(36, 37)
	Phospholipase	Phospholipase A2		(36, 37, 43, 44)

*Modified from Dzobo et al. (39)*.

*NH_4_OH, ammonia hydroxide; Ca(OH)_2_, calcium hydroxide; NaOH, sodium hydroxide; Mg(OH)_2_, magnesium hydroxide; SDS, sodium dodecyl sulfate; SDC, sodium deoxycholate; CHAPS, 3-[(3-cholamidopropyl)dimethylammonio]-1-propamesulfomate; SB, sulfobetaine; EDTA, ethylenediaminetetraacetic acid; RNase, ribonuclease; DNase, deoxyribonuclease*.

### Scaffold-Free Bioprinting

Scaffold-free bioprinting depends on autonomous self-assembly of the tissue as it develops. Autonomous self-assembly relies on the concept that tissues do not require a template or scaffold because they possess innate mechanisms to produce surrounding tissue architecture. This approach attempts to replicate embryonic environmental and structural development by enabling cells to assemble autonomously (23). During scaffold-free bioprinting, prefabricated multicellular building blocks such as cell pellets, spheroids, or tissue strands are utilized to generate 3D constructs. These “building blocks” are printed at high cell densities, fusing together and releasing the desired ECM components of the tissue (22). This cell friendly approach avoids the use of exogenous material, ultimately reducing toxicity, improving cell viability, increasing cell-to-cell interactions and reducing the length of post-bioprinting maturation when compared to scaffold-based bioprinting. However, it requires higher cell densities limiting printer selection, possesses low scalability, and lacks mechanical integrity due to the absence of scaffold or physical support (24).

Cell pellets are concentrations of cells generated via centrifugation or other gravitational techniques. The advantage of this technique is that it does not require a sophisticated system to use; however, it is limited in its ability to circulate growth media and oxygen during tissue development. As a result, there is marked reduction in cell viability when using pellet-based scaffold-free bioinks (26). Tissue spheroids are cellular aggregates, which can be used as building blocks in bioprinting applications. Several techniques have been employed to generate tissue spheroids; the most common utilizes micro-molded non-adhesive hydrogels to facilitate spheroid production. Although this method relies on sophisticated technologies and requires a great deal of skill, it enables intense cell-to-cell interaction and recapitulates other physiological conditions such as nutrient and oxygen diffusion gradients as well as a pH that closely resemble native tissue (26, 30). Cylindrical neo-tissue strands represent the newest strategy for scaffold-free bioprinting. Neo-tissue strands are generated by injecting a high number of cells into a supportive, tubular, semi-permeable structure that allows for nutrient and gas exchange. Following development of these neo-tissue strands, the tubular structure degrades, leaving perfectly cylindrical tissue strands that are subsequently loaded into a custom extrusion based bioprinter nozzle where it is subsequently printed into the desired structure (45).

Following preparation, these bioinks are loaded into bioprinting ink cartridge and printed using extrusion based bioprinting. Because of the lack of innate structure in cell pellets and spheroid bioinks, they are printed into a structural mold that aids in cellular organization and helps facilitate intracellular interactions and ECM development. This requirement limits their scalability as a result of marked increases in processing time pre-bioprinting (26). Unlike spheroids or cell pellets, cylindrical neo-tissue strands do not require a mold, as they possess an innate structural integrity. This allows them to be printed directly following their generation, making scalability more feasible, and showing promise for future scaffold-based bioprinting approaches (45).

### Synergetic Approach

To overcome limitations associated with scaffold-based and scaffold-free approaches, alternative techniques combining these methods have been developed. These approaches utilize microscaffolds and spheroids or other hybrid constructs to facilitate tissue engineering. One example of this synergistic approach is the “lockeyballs” technique. This technique uses the combination of micro-scaffolds and spheroids; whereby microscaffolds enable individual spheroids to fuse together while providing mechanical integrity (46). These approaches have shown promising results in facilitating cell-to-cell contact as well as improving the vascularization and structural integrity of bioprinted tissues (22).

### Additional Bioink Characteristics

Bioinks can be further subdivided based on the sources they were derived from, each of which has their own inherent advantages and disadvantages (31, 47, 48). Although the bioink's source is one of the most critical elements, other factors including its physical properties, ionic charge, and crosslinking can significantly affect 3D bioprinting. Bioink properties are chosen to complement the printer type used as described in Supplementary Table 1 (26).

## Mechanism of Bioprinters

**Figure 3:**
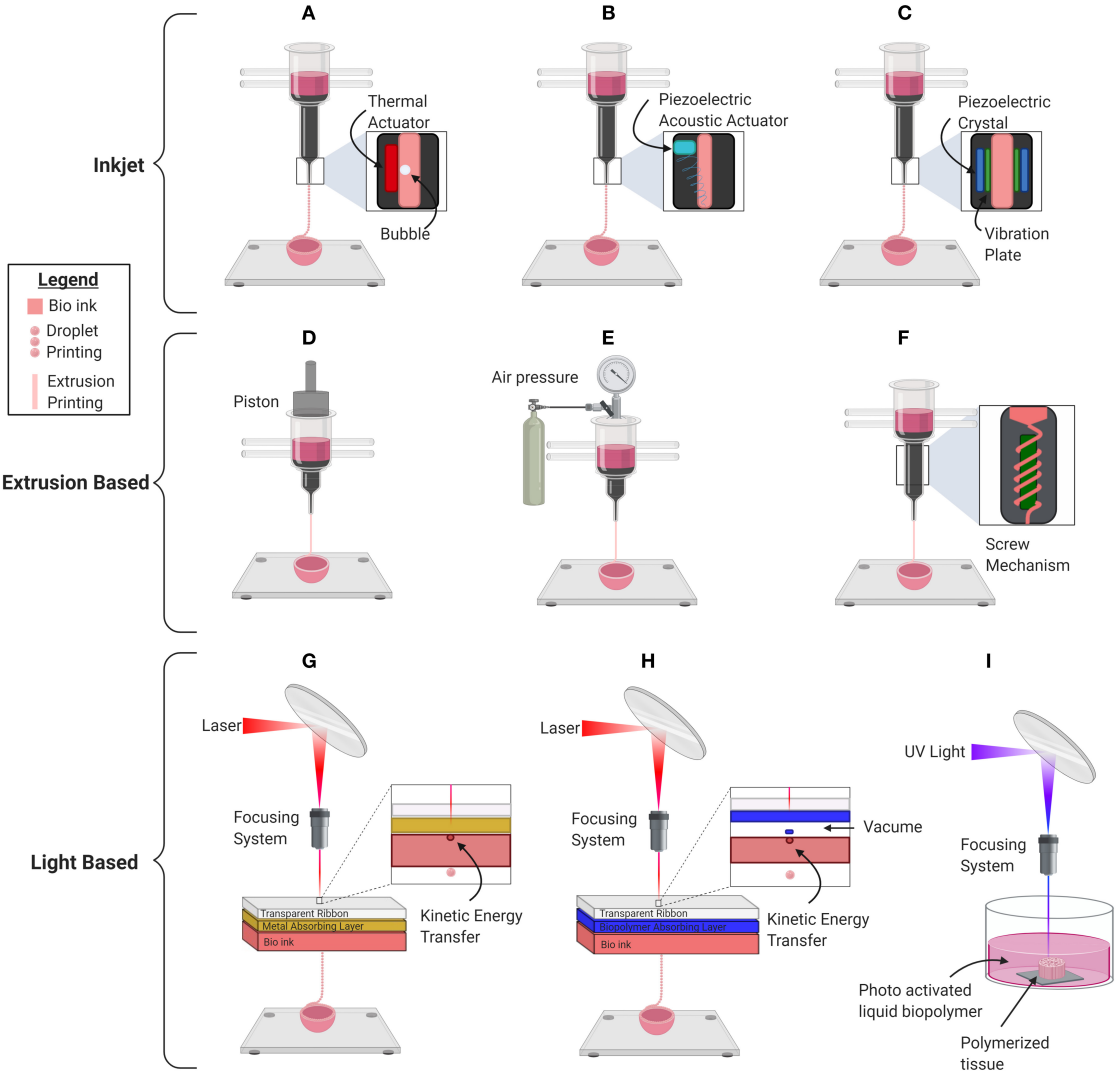
Schematic depiction of the basic mechanical inner workings of the current commercially used major bioprinting technologies for printing organs, organoids, and other biological material. **(A)** Thermal inkjet printer. **(B)** Piezoelectric mechanical inkjet printer. **(C)** Piezoelectric acoustic inkjet printer. **(D)** Piston-based extrusion printer. **(E)** Pneumatic extrusion bioprinter. **(F)** Screw based extrusion printer. **(G)** Laser Induced Forward Transfer (LIFT). **(H)** Matrix-Assisted Pulsed Laser Evaporation (MAPLE). **(I)** Stereolithography Bioprinter.

The selection of bioink as well as a scaffold-based vs. scaffold-free approach is significantly influenced by the type of bioprinter that is going to be used. There are four major types of bioprinters: inkjet droplet, extrusion, laser droplet, and stereolithography (Figure 3, Table 2). In inkjet bioprinting, surface tension holds the bioink at the nozzle of the printer and several strategies are used to force droplets out in a controlled fashion. Thermal inkjet printers apply bursts of 200°C energy, lasting ~2 microseconds (51, 58, 59). The burst of heat rapidly develops a bubble which forcibly ejects the biomaterial in a dropwise fashion (51, 59, 60). Mechanical piezoelectric inkjet printers have a charge applied to a piezoelectric crystal, causing it to contract, forcing a vibration plate to apply mechanical pressure to the nozzle and evoke droplet extrusion (51, 58). In contrast, acoustic piezoelectric inkjet printers use a piezoelectric crystal to create acoustic waves of energy that break the surface tension of the bioink air interface at the end of the nozzle (61).

**Table 2:** Specification of different classifications of bioprinters, their advantages, disadvantages, and required bioink characteristics.

**Printer type**	**Printer subtype**	**Resolution^a^ capabilities**	**Viscosity (mPa·s)**	**Max cell densities (cells/mL)**	**Cell** **viability^**b**^**	**Bioink required characteristics^**c**^**	**Advantages**	**Disadvantages**	**References**
Inkjet (Figures 3A–C)	Thermal (Figure 3A)	High	3–30	10^6^	80%	- Low viscosity - Rheopectic behavior - Non-fibrous nature - Medium surface tension - Rapid gelation kinetics	- High resolution - Multiple materials	- Low cell density - Thermal stressors - Sheer force stressor	(49–51)
	Piezoelectric Mechanical (Figure 3B)	High	3–30	10^6^	80%	- Low viscosity - Rheopectic behavior - Non-fibrous nature - Medium surface tension - Rapid gelation kinetics	- High resolution - Multiple materials	- Low cell density - Sheer force stressor	(49, 51)
	Piezoelectric Acoustic (Figure 3C)	Medium	3–30	10^6^	95%	- Low viscosity - Rheopectic behavior - Non-fibrous nature - Medium surface tension - Rapid gelation kinetics	- High cell viability - Multiple materials	- Lower resolution than other inkjet printers - Low cell density	(49, 51)
Extrusion based(Figures 3D–F)		Low	Up to 6 × 10^7^	10^8^	40–97%	- Shear thinning - Thixotropic behavior - Low surface tension - Low adhesion - Rapid gelation - Shape retention	- High cell densities - Ability to print scaffold free spheroids - Multiple biomaterials from separate nozzles	- Low resolution, cell viability and printing speed, nozzle clogging at high viscosities	(2, 52, 53)
Laser Induced Forward Transfer(Figure 3G)		High	1−300	10^8^	90%	- Adhesion to the intermediate layer - Low surface tension - Viscoelasticity - Absorption kinetic energy - Rapid gelation	- High cell densities - High cell viability - Medium Viscosity	- Expensive - Exposure to toxic particles and radiation, long term damage - Single bio-material at a time, low scalability - Not ECM relevant viscosity	(54–56)
Stereolithography(Figure 3I)		High		10^6^	85%	- Undergo photopolymerization - Use of light absorber - Use of photo-initiators with low toxicity - Stability and high-mechanical strength - Retention of uniform cell distribution	- Excellent for bioprinting scaffolds -Very high resolution	- Requires photo-activated polymers - DNA damage from exposure to UV rays withunknown long-term effects	(56, 57)

a *Resolution refers to the ability of the printer to precisely place biomaterial in a controlled manner and is dependent on the droplet size, and speed of the printer. ^b^Cell viability refers to the percentage of cells alive 24 h post printing. ^c^Column modified from Hospodiuk et al. (26)*.

Extrusion-based bioprinters have the capability of printing cell dense, viscous bioinks, using one of three main mechanisms to continuously force viscous biomaterial out of the nozzle in a controlled manner. The pneumatic mechanism applies air pressure to the surface of the bioinks. The mechanical mechanism applies mechanical force to the surface of the bioink using a piston, and the screw-based mechanism applies a rotational force to continuously extrude bioinks (52, 53). Despite the possibility of nozzle clogging and low printing resolution, the main advantage of this scalable technology is its ability to print high, biologically relevant, viscosities allowing the use of scaffold-free spheroid bioinks (52).

There are multiple variations of laser printers, all of which use the laser-induced forward transfer (LIFT) mechanism. LIFT avoids nozzle clogging by using a high-powered laser directed through a transparent glass into an energy-absorbing layer of gold, titanium, or other metal (62, 63). When the laser is pulsed, an energy-absorbing layer transfers energy to the bioink, which facilitates its release in a highly controlled manner (63). Modifications to LIFT have been implemented to reduce the exposure of bioinks to photons and toxic particles to maintain the viability of its contents. Thicker (100 nm) energy-absorbing layers protect the biomaterial from photo exposure, whereas matrix-assisted pulsed laser evaporation (MAPLE) technology uses a biopolimer matrix to transfer kinetic energy, which further reduces the exposure of bioinks to toxic particles (56, 64). Although laser printers are expensive and lack scalability, they are capable of printing cell dense bioinks at a high resolution.

Finally, stereolithography bioprinting uses a pool of liquified cell laden biopolymer that is photoactivated by UV light. Precise movement of the UV light by a computer causes macromolecules to crosslink in a highly controlled manner and stimulates the development of tissue architecture (65). Stereolithography bioprinting offers high resolution as polymerization can be initiated precisely using a single photon. In addition, this nozzle free approach avoids the issue of nozzle clogging. However, the bioinks are limited because they are required to have the ability to photo polymerize and there is a risk of damaging the cell DNA due to UV light exposure.

3D bioprinting technologies produce naïve tissue constructs via the outlined processes. However, regardless of which 3D bioprinting mechanism is used, the novel tissue must generally undergo further maturation before being ready for transplantation.

## Tissue Maturation

Bioprinted constructs are matured under tightly regulated temporal and environmental conditions. This is frequently accomplished using a bioreactor. Bioreactors provide physical and biochemical signals to direct *in vitro* tissue development. Several bioreactor systems have been developed; these include static culture, spinner flask, and perfusion systems (66). Bioreactor systems regulate temperature, pH, CO_2_ concentration, hydrostatic pressure and shear stress using computational methods as described elsewhere (66, 67). This allows them to reproduce the physiological environment of the intended location of transplantation. Biological factors such as temperature and CO_2_ concentration influence the metabolism and growth of developing tissues, whereas mechanical factors such as pressure and shear stress are critical in establishing appropriate tissue function (66). This is especially true for cartilage development. Daily activity subjects' cartilaginous tissues to periods of stress and compression, which lead to temporal increases in hydrostatic pressure. Hydrostatic pressure prompts cartilaginous cells to retain synovial fluid and increase both proteoglycan and collagen synthesis, which helps strengthen surrounding architecture and further tissue development (66).

An alternative approach to conventional bioreactor systems is *in situ* bioprinting. *In situ* bioprinting differs from conventional bioprinting techniques as tissues are directly printed into the desired location of transplantation within a living host (67). Common techniques involve the use of robotic arms or handheld devices such as the biopen to facilitate *in situ* deposition. The process eliminates the need to recapitulate the native tissue environment *in vitro* and, instead, relies on signals from adjacent tissue to act as an “*in vivo* bioreactor” and direct construct development. By utilizing native signaling, *in situ* bioprinting greatly reduced risks associated with poor integration of *in vitro* constructs (67). This approach also eliminates possible damage during implantation of delicate *in vitro-*derived constructs (67). In addition, due to resolution limits of imaging techniques, the shape/size of an *in vitro*-derived construct may be incompatible with the target defect, where *in situ* bioprinting would avoid this issue (67). However, *in situ* bioprinting requires a very high cell number, is expensive, and incompatibility with host tissues may lead to aberrant tissue development or severe immunological reactions (67). Though this method is currently in its infancy, there have been some studies investigating its use in the generation of skin, cartilage, and bone (68–70). One such proof-of-concept study looked to repair osteochondral defects in bovine femoral condyles. Using a demineralized bone matrix paste and an alginate hydrogel, a robotic arm printed a dome-shaped bone plug covered by a cartilaginous cap. These printed constructs had low mean geometric errors and closely mimicked the original pre-defect contour of the femoral condyle (68).

## 3D Bioprinted Tissue and Organ Constructs

3D bioprinted constructs have been utilized to investigate novel drug therapies, develop patient-specific treatment plans, and study complex physiological processes (71). However, for some, the ultimate goal of this technology is to fabricate fully functional organs for *in vivo* application. 3D bioprinted structures could replace diseased or damaged organs, alleviate strains associated with finding appropriate donor organs, and minimize immune complications and/or anatomical incompatibilities that can arise from allogenic transplant (6). Recent advances in human research have led to the production of 3D bioprinted cardiac patches used to treat myocardial infarctions in rat models, bioprinted corneal constructs shown to successfully integrate with host porcine tissue, and many other promising preliminary studies, as outlined in Supplementary Table 1 (72, 73). Despite the large amount of research in human medicine, veterinary studies remain limited. Most studies have focused on the *in vitro* rather than *in vivo* capabilities of bioprinting. However, human research efforts have frequently used animal models in their investigations (Supplementary Table 1). This has provided veterinary researchers with information regarding the efficacy and safety of bioprinted tissues, challenges associated with its *in vivo* integration, and provided them with a foundation for future veterinary research.

3D bioprinting has been utilized to generate bone, cardiovascular, cartilage, corneal and neural constructs in a variety of animal species (as outlined in Supplementary Table 1) (74). However, much of this research has focused on the generation of either cartilage or bone. 3D bioprinted articular cartilage generated from equine, bovine, cuniculus, and porcine species, have demonstrated appropriate *in vivo* mechanical properties, cell densities, and collagen remodeling (8–10). Chondrocyte-embedded constructs implanted into cartilage defects of rabbit ears were shown to successfully integrate with surrounding cartilage and facilitate complete regeneration of the defect (7). When whole-segment tracheal constructs 3D bioprinted using autologous auricular chondrocytes were transplanted into goats with tracheal defects; recipient goats exhibited increased survival time, and bioprinted constructs possessed greater compressive strength than native tissue (11). Veterinary researchers have had additional success bioprinting osseous tissue. Osteochondral constructs produced using cellular components derived from equine, porcine, and cuniculus species have exhibited appropriate *in vivo* biomechanical properties, high cell viability and osteochondral differentiation (75–78). Osteochondral constructs implanted into a goat articular defect demonstrated biocompatibility with host tissue and facilitated complete regeneration of trabecular bone (79). The perceived focus of veterinary research efforts on articular defect repair is likely due to the considerable prevalence of these injuries in veterinary medicine, particularly among horses, and the less complicated nature of printing these constructs. Cartilage is devoid of vascular and neuronal tissues and, as such, challenges associated with bioprinting vasculature is avoided.

Researchers have recently begun investigations into the benefit of bioprinting in the repair of cardiovascular, optical, and nervous tissues. 3D bioprinted vascular grafts, comprised of autologous mesenchymal stem cells printed onto conventional artificial grafts, were implanted into canine bilateral carotid and femoral arteries. Those that received the bioprinted grafts showed increased endothelialization and decreased inflammation relative to a control group that received artificial grafts alone (80). Microsteriolithography was utilized to generate an artificial limbus structure using rabbit limbal fibroblasts and endothelial cells. This structure was implanted into a rabbit *ex vivo* wounded cornea model where it proliferated and successfully formed multilayered epithelium (81). 3D bioprinted nerve conduits were generated using spheroids comprised of canine-derived dermal fibroblasts and implanted into the ulnar nerves of dogs possessing a deliberate 8 mm defect. Ten weeks following surgery, the printed construct successfully stimulated neural regeneration. Nerves had bridged the construct, and there was a higher number of thin, mature myelinated axons present relative to a control group consisting of dogs that did not receive a neural graft (82). As 3D bioprinting research persists and its technology evolves, future veterinary research efforts investigating the feasibility of 3D bioprinting to generate safe, functional tissue constructs are likely to follow. Though veterinary 3D bioprinting research is currently in its infancy, it possesses significant potential for regenerative medicine and veterinary practice.

## Potential in Veterinary Medicine

Although there are currently few veterinary applications of 3D bioprinting, its prevalence is likely to follow a similar trend to 3D printing of non-biological material. 3D printing has been utilized in the veterinary field to generate 3D surgical models, produce physical aids for veterinary teaching, and to generate personalized implants and prosthetics for companion animals (3, 83). In addition, researchers have begun developing low-cost bioprinting technologies. Yenilmez et al. (84) generated a hybrid droplet-and-extrusion bioprinter with high-throughput and high-resolution capacities that costs $1,400 which is significantly cheaper than commercially available bioprinters that range from $10,000 to over $200,000 (84). Though additional costs associated with the maintenance and use of bioprinters remain high, increased affordability and subsequent veterinary use will likely follow as these technologies continue to evolve.

**Figure 4:**
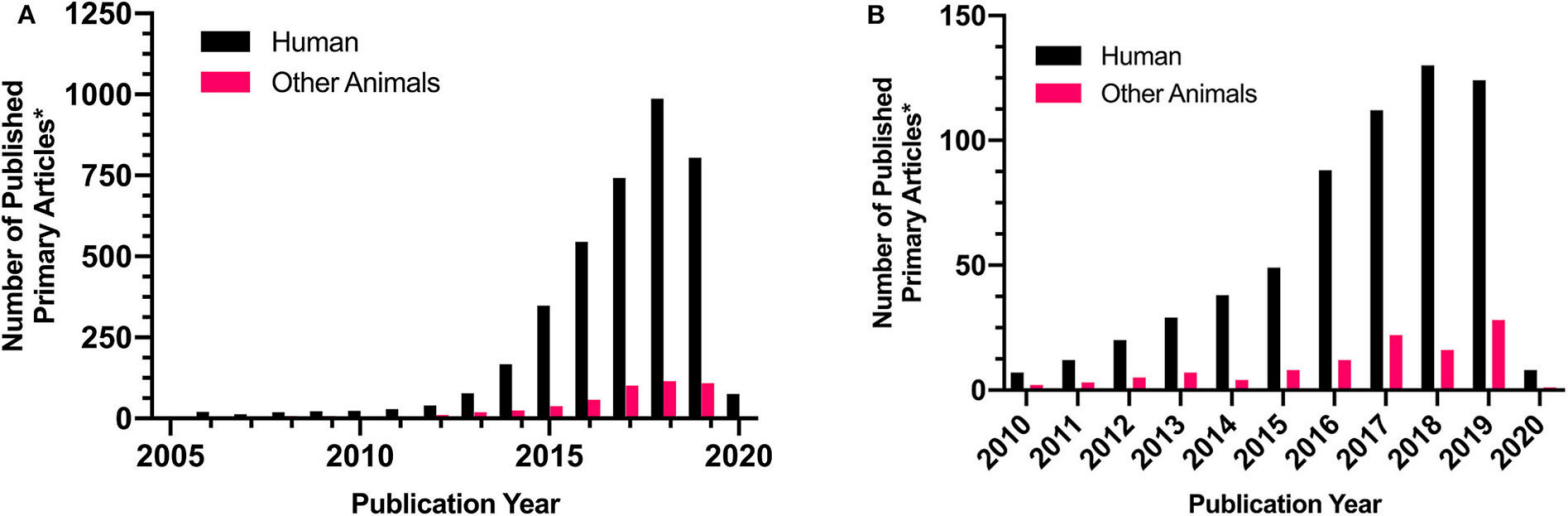
Representative comparison of human and veterinary research articles in the involving “3D Printing” or “bioprinting.” Demonstrates the incidence of primary research on **(A)** 3D printing or **(B)** bioprinting in human and veterinary research Comparison Research article counts were generated via pubmed targeted searches. Searches were conducted on 6/24/2020 with the following keywords: **(A)** “Human+3D Print” and “Other Animals+3D Print”, duplicated and review articles were excluded, and the following species were included into the yearly counts: “Canine,” “Bovine,” “Equine,” “Rabbit,” “Goat,” “Porcine.” This was repeated following the initial searches: **(B)** “Human+bioprint” and “Other Animals+bioprint” with the same exclusion criteria. This is not an exhaustive literature search but is believed to be representative of trends in scientific literature.

Most research efforts in the veterinary field have focused on its *in vitro* application. However, recently there has been a growing number of publications on its utility in veterinary science (Figure 4). 3D bioprinted bovine colon organoids have been established for use in agri-biotechnological and pharmaceutical applications (85). Further development of these technologies could yield high-throughput methods to more accurately assess4 bioactive compounds while reducing the frequency of animal testing (85). Baird et al. (77) utilized 3D printed thermoplastic scaffolds and equine iPSCs to investigate the effectiveness of bioprinting technologies in equine fracture repair and the treatment of bone defects (77). Deposition of iPSCs onto thermoplastic scaffolds enabled successful iPSC differentiation which resulted in osteoblast formation and subsequent bone mineralization. Furthermore, research efforts directed at the application of 3D bioprinting in human medicine have tended to utilize animal models and/or animal-derived stem cells to investigate the capacities of 3D bioprinting technologies (Table 2). Testing 3D bioprinted tissues in animals may provide valuable information for both human and veterinary medicine and help expedite veterinary translation. Several proposed veterinary applications include canine bone remodeling, equine cartilage repair, and more accurate disease modeling for various species (3). These technologies could provide novel opportunities in tissue engineering and possibly organ transplantation into various animal species.

## Barriers to Translation

Although 3D bioprinting possesses significant clinical potential, it must overcome several technological, regulatory, and ethical challenges prior to translation. Current bioprinters are faced with tradeoffs between their ability to print at a high resolution or biologically relevant viscosities. This has made it difficult for scientists to generate complex tissue structures at an anatomical scale that are capable of long-term survival (56). In addition, 3D bioprinting has yet to efficiently incorporate tissue vasculature. This has limited the ability of generated tissues to successfully engraft and integrate with existing vascular networks (86). Strategies involving decellularization in conjunction with 3D bioprinting have improved the vascularization of these tissues; however, further work on the ability of these organs to sustain long-term vascular integration remains incomplete (39).

Accessibility of 3D bioprinting technologies will largely depend on their commercialization and regulation. Currently, 3D bioprinting is not covered under any established regulatory framework. The governments of Canada, the United States of America (USA) and European Union (EU) have produced guidance documents on the manufacturing of 3D printing technologies, but none include any provisions pertaining to 3D bioprinting (87). As a result, there exists significant ambiguity regarding the classification and patentability of these technologies which will have a significant influence on future developmental efforts and accessibility (5, 88). If deemed patentable, companies would be able to form market monopolies and restrict affordability and accessibility. However, current regulations deem methods that rely on the destruction of human embryos unpatentable and both ethical provisions and medical treatment exemptions exist across countries jurisdictions to prevent monopolization in medical markets (88, 89). Though classification and patentability are currently ambiguous, it is believed over the next few years, national guidelines will be established.

## Ethical Considerations

The emergence of 3D bioprinting technologies has stimulated ethical discussions pertaining to their use. 3D bioprinting generally requires the use of stem cells in bioink formulations including embryonic, adult stem cells, and/or induced pluripotent stem cells. Stem cell acquisition has a history of ethical and political controversy as discussed above (5). Increased use of 3D bioprinting technologies may further exacerbate controversy as a result of increased stem cell demand.

Advancements in 3D bioprinting may provide opportunities for tissue and organ transplantation in veterinary medicine. Organ transplantation is a major procedure that has drastic, long-lasting effects on the recipient. Thus, it is important to consider the ethical responsibility of manufacturers and veterinary professionals in terms of donor and recipient selection. To ensure the safety of the recipient, quality assurance measures must be employed at every stage of the bioprinting process. Clinical trials will likely have to evolve from the transplantation of tissue and smaller organ structures before transplantations of larger organ structures can be attempted. Educational programs must be developed to ensure that veterinarians can provide owners with an accurate understanding of these technologies (90). Although, it can still be argued that the safety of the recipients cannot be adequately ensured with any current processes involved in clinical translation (87). Therefore, it will be important to involve veterinarians, veterinary researchers, public health officials, regulatory authorities, and other community stakeholders in the development of new regulatory measures.

Increased accessibility to 3D bioprinted tissues could also affect public behavior and disturb human-animal relations. For example, if 3D bioprinted human organs become easily accessible, individuals may be more likely to perform more harmful activities, such as smoking, excessive drinking, and/or drug use, with less fear of the repercussions. If so, it may be reasonable to assume that this lapse in judgment may influence their behavior toward animals (91). 3D bioprinting could lead to conflicts between animal welfare and veterinary medical technology as in the case of double-muscled Belgium Blue Cattle. These cattle were produced through targeted breeding programs resulting in removal of the bovine myostatin gene (*MSTN*), a gene involved in the regulation of skeletal muscle development. The goal was to produce cattle with a greater meat yield and a higher percentage of high-value cuts. However, it inadvertently led to health issues such as dystocia, reduced calf fertility, and reduced calf survival, thereby negatively affecting the welfare of the cattle (92). Similarly, 3D bioprinting could incentivize owners to perform augmentative treatments and/or numerous tissue replacements, particularly on performance animals such as racehorses, to further the longevity of their animal's performance. This could threaten the quality of life of these animals and prolong suffering prior to compassionate euthanasia. Thus, education programs will need to be developed to inform stakeholders on the risks and limitations associated with bioprinting modalities. Though 3D bioprinting may help bolster the capacities of both veterinary and human medicine, if misused, it could drastically affect patient well-being.

## Conclusion

3D bioprinting is a rapidly emerging industry that could benefit both human and veterinary medicine. Advances in bioprinting have led to the production of higher resolution bioprinters, improved vascularization of printed tissues, and the generation of *in vitro* and *in vivo* tissue models. As these technologies have continued to evolve, we have seen an increase in research efforts directed at veterinary application. Current limitations such as the inability of bioprinters to print at both high resolution and viscosity, bioinks to maintain cell viability during printing, and the successful vascular integration of printed tissues have impeded clinical translation. Further, patentability and regulation of this technology provide additional challenges that can strongly influence accessibility to these technologies. However, the biomedical and economic potential of 3D bioprinting will inevitably lead to solutions to these issues. With increased use and development, 3D bioprinting is likely to emerge as a powerful tool for regenerative medicine in the veterinary sciences. It could improve the treatment of several veterinary conditions such as diabetes, cancer, and musculoskeletal injuries, and 3d bioprinted organ transplantation may 1 day become commonplace.

## Author Contributions

CJ: abstract, introduction, tissue maturation, potential in veterinary medicine, barriers to translation, ethics, conclusion, editing and togethering sections together, and Figure 4. PK: mechanism of bioprinters, general editing, and Figures 1, 3, Table 2. SO: cellular component of bioink and parts of 3D bioprinting approach and bioink selection. CW: 3D bioprinted tissue and organ constructs and Supplementary Table 1. D'AK: dECM component of scaffold-based bioprinting, Figure 2, and Table 1. KR and TK: advisory roles and editing. All authors: contributed to the article and approved the submitted version.

## Conflict of Interest

The authors declare that the research was conducted in the absence of any commercial or financial relationships that could be construed as a potential conflict of interest.
