# Supplementary material for: A Review of Recent Advances in 3D Bioprinting With an Eye on Future Regenerative Therapies in Veterinary Medicine
Source: Front Vet Sci. 2021 Feb 16;7:584193. doi: 10.3389/fvets.2020.584193 (PMC7921312; doi:10.3389/fvets.2020.584193)
Supplement: Supplementary file 1 [file Table_1.DOCX]

**Supplementary Material**

**Supplemental Table 1. Examples of *in vitro* and *in vivo* studies that utilize 3D bioprinting techniques to generate tissue constructs.**

| **Tissue Type** | **Species** | **Cell Type** | **Main Outcomes** | **Reference** |
| --- | --- | --- | --- | --- |
| Adipose | Human | ASCs | Human lipoaspirate-derived adipose tissue containing ASCs showed high printability, survived 3D bioprinting and engraftment into mice in vivo, and displayed macroscopic and microscopic evidence of vascularization | (77)Saljo *et al.* 2020 |
|  |  | ASCs | ASC-derived spheroids were bioprinted onto a scaffold and showed multilocular microvacuoles, successful differentiation toward mature adipocytes and were able to assemble at random | (78)Colle *et al.* 2020 |
| Bone | Caprine | BMSCs | BMSCs were seeded into 3D bioprinted biomimetic osteochondral constructs. Constructs were implanted into a goat articular-defect model and showed good biocompatibility and regeneration of the trabecular bone | (73)Zhang *et al.* 2017 |
|  | Equine | iPSCs | 3D printed polymer was used as a scaffold for in vitro osteoblast differentiation from equine-derived iPSCs. Differentiated cells produced a mineralised matrix, synthesized alkaline phosphatase, and expressed osteoblast-associated genes and proteins. | (69)Baird *et al.* 2019 |
|  | Cuniculus | NPCs*, AFCs | Rabbit intervertebral discs were precisely replicated using 3D bioprinting. Constructs showed appropriate porosity, compressive stiffness, and mechanical properties. | (70)Uden *et al.* 2015 |
|  | Porcine | MSCs | Demonstrated that both pre-differentiated and undifferentiated MSCs could be 3D bioprinted without any cell impairment or negative affects to MSC differentiation. Constructs demonstrated alkaline phosphatase activity, indicating osteogenic differentiation | (71)Gruene *et al.* 2011 |
|  |  | BMSCs | Looked to investigate BMSC viability and direction of cell fate following 3D bioprinting of cylindrical constructs. Fine-tuning of bioink stiffness and composition resulted in constructs that demonstrated high cell viability and differentiation of BMCs into distinct cell lineages. | (72)Freeman and Kelly, 2017 |
|  | Human | hMSCs, HUVECs | 3D bioprinted scaffolds containing hMSCs and HUVECs generated an in vitro bone model that demonstrated osteogenesis and robust vascularisation | (80)Chiesa *et al.* 2020 |
|  |  | MSCs | A novel bioink was combined with MSCs and calcium phosphate cement to generate 3D bioprinted bone-like constructs that displayed appropriate cell proliferation and differentiation. | (81)Ahlfeld *et al.* 2020 |
|  |  | hTMSCs | Multilayered osteochondral constructs were generated via 3D bioprinting and were inserted into rabbits with femoro-patellar defects and were left for 8 weeks. Constructs showed osteochondral and neocartilage regeneration, displayed appropriate chondrogenic markers, and high histological scores | (82)Shim *et al.* 2016 |
| Cardiovascular | Canine | MSCs | Vascular grafts were produced by 3D bioprinting a 3 layered scaffold that contained canine-derived MSCs. Constructs were implanted into the bilateral carotid and femoral arteries of 8 dogs. Cell-derived constructs showed significantly more endothelialisation and less inflammation compared to control group. | (74)Jang *et al.* 2020 |
|  | Porcine | Aortic VICs, Aortic root sinus SMCs | Porcine aortic VICs and human aortic root SMCs were encapsulated into separated hydrogels and were used in conjunction to produce a 3D bioprinted aortic valve conduit. Constructs demonstrated adequate biomechanical properties and high cell viability, spreading, and phenotype retention during in vitro culture. | (83)Duan *et al.* 2013 |
|  | Human | iPSCs, CMs, FBs, ECs | Cardiac patches were produced from mixed cell spheroids via 3D extrusion bioprinting. Patches were implanted into rats to repair myocardial infarctions (MIs). Rats treated with the cardiac patch had a 100% survival rate, where control rats had an 83.3% survival. Rats treated with the cardiac patch had significantly less scarring, a significantly increased number of blood vessels, and better cardiac function compared to that of the control rats. | (84)Yeung *et al.* 2019 |
|  |  | iPSC-CMs, FBs, HUVECs | Seeded mixed cell spheroids into a mold-based apparatus that allowed for the rapid assembly of cardiac tissue. Following removal from the mold, cardiac patches began to beat spontaneously after 24 hrs, secreted paracrine factors, were appropriately vascularised and electrically conductive. | (85)Matsushita *et al.* 2018 |
| Cartilage | Bovine | Chondrocytes | Chondrocytes were embedded into collagen bioinks and square cartilage constructs were generated via 3D bioprinting. Constructs showed >90% cell viability and remodelling of the collagen matrix over 14-day in vitro culture. | (9)Diamantides *et al.* 2019 |
|  |  | Chondrocytes | Looked to investigate the effects of different hydrogel sterilisation methods on cytocompatability in 3D bioprinted constructs. Chondrocyte-laden 3D bioprinted constructs that were UV-irradiated demonstrated high cell survival | (86)Hodder *et al.* 2019 |
|  |  | Chondrocytes | Overlayed bovine chondrocytes on a 3D bioprinted articular cartilage construct. Constructs demonstrated 7-day cell viability at 91% compared to the positive control | (87)Kesti *et al.* 2015 |
|  | Caprine | Chondrocytes | Whole segment tissue-engineered tracheas were 3D bioprinted using autologous auricular cartilage cells. Constructs demonstrated a more robust compressive strength than native trachea and showed maturation of cartilage-like tissue. Constructs were transplanted in vivo into a goat tracheal defect model. Treated goats had significantly longer survival times than control goats with autologous tracheal transplants. | (8)Xia *et al.* 2019 |
|  | Equine | MSCs | Equine-derived MSCs were embedded into a hydrogel and were bioprinted onto an organized fiber scaffold generated by melt electrowriting. Constructs demonstrated that mechanical integrity, cell viability and chondrogenic differentiation was not compromised. | (10)de Ruijter *et al.* 2019 |
|  |  | ACPCs, MSCs | A 3D bioprinted articular cartilage construct was created from a bioink laden with equine derived ACPCs and MSCs. Constructs supported neo-cartilage synthesis in vitro and demonstrated compressive stiffness seen in immature cartilage. | (79)Levato *et al.* 2017 |
|  | Cuniculus | Chondrocytes | Chondrocyte-embedded cell printed structures were implanted into cartilage defects in rabbit ears and were left for 3 months. Constructs facilitated complete cartilage regeneration and completely integrated into host cartilage. | (7)Park *et al.* 2017 |
|  | Porcine | MSCs, Chondrocytes | Cell-laden hydrogels were 3D bioprinted onto scaffold microchambers which resulted in organised arrays of cellular spheroids. Constructs mimicked the native structure and biomechanical properties of articular cartilage | (88)Daly and Kelly, 2019 |
|  |  | FPSCs | FPSCs were embedded into a hydrogel that had been functionalised with meniscus ECM and the resulting bioink was 3D bioprinted to generate a meniscus construct. Construct supported meniscal phenotypes and did not negatively affect cell viability. | (11)Romanazzo *et al.* |
|  | Human | hASCs | Developed an in-situ 3D bioprinting approach using hASCs and hydrogel to promote articular cartilage regeneration and repair | (89)Duchi *et al.* 2020 |
|  |  | MSCs | 3D bioprinted articular cartilage constructs that were an appropriate thickness and demonstrated paracrine communication between chondrocytes. Further in vivo studies need to be conducted | (90)Henrionnet *et al.* 2020 |
| Corneal | Cuniculus | RLEs, RLFs | RLEs and RLFs were embedded into 3D bioprinted ring scaffolds that mimicked the limbal region of the eye. Cell loaded constructs were placed on an ex vivo wounded cornea rabbit model and demonstrated formation of a multilayered epithelium | (75)Ortega *et al.* 2013 |
|  | Human | hESC-LESCs^k^, hASCs^l^ | Used a combination of cell types and bioinks to bioprint 3 corneal structures. The morphologically and structurally correct constructs were implanted into porcine corneal organ cultures where they attached to host tissue and demonstrated hASC migration. | (91)Sorkio *et al.* 2018 |
| Kidney | Human | iPSCs | Differentiation protocol was developed that allowed for the simultaneous differentiation of all four renal progenitors from iPSCs. Kidney organoids contained all the components of the native kidney and expressed appropriate specialised cell types | (92)Takasato *et al.* 2016 |
|  |  | iPSCs | Using a modified feeder-free protocol from Takasato et al. 2015, generated kidney organoids that were transplanted under the renal capsule of immunocompromised mice for up to 28 days. Resulted in progressive maturation of nephron structures and organoid vascularisation. | (93)van den Berg *et al.* 2018 |
| Liver | Human | iPSC-HPCs | Two-step bioprinting approach that embedded iPSC-HPCs onto a scaffold that mimicked the anatomical structure of hepatocytes. 3D printed hepatic model demonstrated increased phenotypic and functional enhancements over several weeks of in vitro culture | (94)Ma *et al.* 2016 |
|  |  | iPSC-HLCs | 3D bioprinted iPSC-HLC spheroids showed increased cell survival, and hepatic and metabolic function compared to single cell constructs. | (95)Goulart *et al.* 2019 |
| Muscle | Human | hMPCs, hNSCs | 3D bioprinted human neural-skeletal muscle constructs showed improved myofiber formation, long-term survival, neuromuscular junction formation in vitro. Constructs were implanted into rats and facilitated rapid innervation and matured into organised muscle tissue | (96)Kim *et al.* 2020 |
|  |  | hUCB-MSCs | 3D bioprinted scaffolds filled with hUCB-MSCs improved regenerative processes in rabbits with full thickness rotator cuff tears | (97)Rak Kwon *et al.* 2020 |
| Neural | Canine | Dermal-FBs | Nerve conduits were formed via 3D bioprinting of canine-derived dermal fibroblast spheroids. 8mm nerve conduits were implanted into the ulnar nerve of 6 dogs and were evaluated 10 weeks after surgery. Nerves had successfully bridged the conduit and contained a higher number of mostly mature myelinated axons. | (76)Mitsuzawa *et al.* 2019 |
|  | Porcine | Schwann Cells | Demonstrated that primary Schwann cells can be piezoelectrically bioprinted with no adverse effects. Roughly 90% of cells remained viable and expressed appropriate phenotypes. | (98)Tse *et al.* 2016 |
|  | Human | iPSC-NPCs | Dome-shaped 3D neural construct were bioprinted using iPSC-derived NPCs and microspheres. Constructs exhibited appropriate neural markers and demonstrated that this method can promote the differentiation of neural tissue | (99)Sharma *et al.* 2020 |
|  |  | hNSCs | 3D bioprinted neural constructs had functional neurons, supporting microglia and were responsive to electrical stimulation. | (100)Tomaskovic-Crooke *et al.* 2020 |
|  |  | NSCs | 3D bioprinted spinal cord constructs comprised of a 3D scaffold and NSCs showed that they could promote the repair of spinal cord injuries in a rat model. | (101)Jiang *et al.* 2020 |
| Pancreatic | Human | HUVECs | Generated a protocol for preparing a pancreatic tissue-derived decellularized extracellular matrix (pdECM) bioink. Pancreatic tissue constructs were then created via micro extrusion-based 3D bioprinting methods | (102)Kim *et al.* 2019 |
|  |  | iPSCs | Generated islet organoids using a multilayer microfluidic chip device by initial embryoid body (EB) formation followed by pancreatic induction differentiation. Exhibited appropriate morphology, multicellular complexity, and enhanced expression of B-cell associated genes, insulin secretion levels, and the ability to appropriately respond to glucose | (103)Tao *et al.* 2019 |
| Skin | Human | NHDFs, HMVECs, NHEK | Bilayered skin grafts were 3D bioprinted onto a deposition plate. Constructs were placed over top of a full thickness wound in athymic mice. Treatment groups showed improved wound contraction and grafts demonstrated appropriate morphology and adequate cell survival | (104)Yanez *et al.* 2015 |
| Tendon | Porcine | ASCs | Porcine-derived ASCs were seeded onto 3D bioprinted cylindrical scaffold-fiber composites. Tendon-mimicking constructs demonstrated significantly higher mechanical performance compared to collagen-glycosaminoglycan scaffolds and displayed high bioactivity | (105)Mozdzen *et al.* 2016 |

ASCs; adipose-derived stem cells, BMSCs; bone marrow-derived stem cells, NPCs*; nucleus pulpous cells, AFCs; annulus fibrous cells, iPSCs; induced pluripotent stem cells, MSCs; mesenchymal stem cells, hMSCs; human mesenchymal stem cells, HUVECs; human umbilical vein endothelial cells, hTMSCs; human turbinate-derived mesenchymal stromal cells, VICs; valve leaflet interstitial cells, SMCs; smooth muscle cells, CMs; cardiomyocytes, FBs; fibroblasts, ECs; endothelial cells, ACPCs; articular cartilage progenitor cells, FPSCs; fat pad-derived stem cells, hASCs; human adipose-derived stem cells, RLEs; rabbit limbal epithelial cells, RLFs; rabbit limbal fibroblasts hESC-LESCs; human embryonic stem cell-derived limbal epithelial stem cells, iPSC-HPCs; iPSC-derived hepatic progenitor cells, iPSC-HLCs; iPSC-derived hepatic-like cells, hMPCs; human muscle progenitor cells, hNSC; human neural stem cells, hUCB-MSCs; human umbilical cord blood-MSCs, iPSC-NPCs; iPSC-derived neural progenitor cells, NSCs; neural stem cells, NHDFs; neonatal human dermal fibroblasts, HMVECs; human dermal microvascular endothelial cells, NHEK; neonatal human dermal keratinocytes
